# Where to Next? Research Directions after the First Hepatitis C Vaccine Efficacy Trial

**DOI:** 10.3390/v13071351

**Published:** 2021-07-13

**Authors:** Christopher C. Phelps, Christopher M. Walker, Jonathan R. Honegger

**Affiliations:** 1 Center for Vaccines and Immunity, Abigail Wexner Research Institute at Nationwide Children’s Hospital, Columbus, OH 43205, USA; Christopher.Phelps@nationwidechildrens.org (C.C.P.); Christopher.Walker@nationwidechildrens.org (C.M.W.); 2 Department of Pediatrics, The Ohio State University College of Medicine, Columbus, OH 43210, USA

**Keywords:** hepatitis C virus (HCV), vaccine, immunity, T cells, B cells, antibodies, chronic infection, clinical trial

## Abstract

Thirty years after its discovery, the hepatitis C virus (HCV) remains a leading cause of liver disease worldwide. Given that many countries continue to experience high rates of transmission despite the availability of potent antiviral therapies, an effective vaccine is seen as critical for the elimination of HCV. The recent failure of the first vaccine efficacy trial for the prevention of chronic HCV confirmed suspicions that this virus will be a challenging vaccine target. Here, we examine the published data from this first efficacy trial along with the earlier clinical and pre-clinical studies of the vaccine candidate and then discuss three key research directions expected to be important in ongoing and future HCV vaccine development. These include the following: 1. design of novel immunogens that generate immune responses to genetically diverse HCV genotypes and subtypes, 2. strategies to elicit broadly neutralizing antibodies against envelope glycoproteins in addition to cytotoxic and helper T cell responses, and 3. consideration of the unique immunological status of individuals most at risk for HCV infection, including those who inject drugs, in vaccine platform development and early immunogenicity trials.

## 1. The Ongoing Need for a Vaccine

Thirty years ago, the discovery of the small, positive-stranded RNA virus responsible for hepatitis C provided the means to halt disease transmission by blood transfusion and fostered optimism that a vaccine may soon follow [1]. To date, however, no vaccine is available, and the hepatitis C virus (HCV) remains a major global public health concern [2]. An estimated 71 million people are currently infected, and nearly 400,000 individuals die each year due to HCV-associated liver fibrosis and carcinoma [2,3]. New infections continue to occur at a high rate, with an estimated 1.7 million occurring in 2015 [3].

More recently, approval of breakthrough oral direct-acting antiviral (DAA) therapies capable of curing over 95% of chronic HCV infections of any genotype has kindled hope that the global HCV pandemic might be ended through treatment [4]. This led the WHO, in 2016, to set a goal to eliminate HCV as a global public health problem by 2030, specifically by treating 80% of those infected and reducing new infections by 90% [5]. However, the incidence of HCV infections has continued to rise in many countries since the introduction of DAAs. In the US, rates of reported and estimated acute infections nearly doubled in the 5 years following the first approval of interferon-free DAA regimens in 2013 [6]. Challenges to HCV elimination by treatment include (1) failure to recognize most infections due to lack of overt symptoms and deficient screening of those at risk [7], (2) increasing difficulty reaching vulnerable populations after initial waves of treatment in the “easiest” patients already engaged in healthcare [8], (3) high costs of widespread screening and treatment [9], (4) emergence of viral resistance [10,11], and (5) importantly, re-infections following antiviral cure among individuals with ongoing exposure [12]. For these reasons, a prophylactic vaccine is urgently needed to complement ongoing treatment programs in order to meet the imperative to eliminate the global public health burden of HCV [13,14].

### HCV Vaccine Feasibility and Development

HCV is recognized as a challenging vaccine target due to its vast genetic diversity and propensity to evade immunity, but several features of its natural history suggest that vaccination may be feasible. First, because the major health complications of HCV such as cirrhosis or hepatocellular carcinoma arise only after years of persistent viremia [15], a successful HCV vaccine would not necessarily be required to prevent infection. Rather, it must induce immune memory responses capable of clearing infection prior to the onset of adverse outcomes. Second, 20–30% of infected individuals spontaneously resolve viremia in the first 6–12 months, and, if reinfected, these individuals rapidly clear viremia in 80% of cases [16]. This supports the notion that induction of protective adaptive immune memory is possible.

These observations have spurred intensive efforts to identify immune mechanisms of natural viral clearance that might guide vaccine design. Numerous lines of evidence point to a central role of cellular immunity in controlling HCV infection. In acute HCV infection, after a 4–12 week incubation period of high-level viral replication, the appearance of highly functional and broadly reactive CD4^+^ T helper cells [17,18] and CD8^+^ cytotoxic T cells [19,20,21] targeting primarily nonstructural proteins of HCV (NS3-NS5) in the peripheral blood correlates with rapid reductions in viral load in subjects that go on to resolve infection [22,23]. Depletion of either CD4^+^ or CD8^+^ cells in a chimpanzee model of reinfection demonstrated that both populations are required for viral clearance [24,25]. Similar initial responses may be detected early in persisting infections, but HCV-specific CD4^+^ T cell populations soon decline and become nearly undetectable [17,26]. This lack of CD4^+^ T cell help is thought to contribute to an impaired HCV-specific CD8^+^ T cell response [24] with reduced cytokine production, cytotoxicity, and proliferative capacity seen in humans with chronic infection [27,28].

The role of humoral immunity in controlling HCV infection has been less clear. Early evidence of viral clearance in seronegative patients and those with agammaglobulinemia, as well as lack of correlation of development of neutralizing antibodies with viral clearance, suggested that antibody responses may be dispensable and of limited value for resolution of infection [29,30,31,32,33]. On the other hand, passive administration of neutralizing antibodies could prevent infection in mouse and chimpanzee models [34,35,36]. Moreover, active vaccination of chimpanzees with envelope glycoproteins to induce humoral responses, while not necessarily resulting in sterilizing immunity, was protective against chronic infection [37,38]. More recent studies found that spontaneous clearance in humans is associated with the early development of antibodies capable of neutralizing autologous circulating viral strains or libraries of viral strains [39,40].

Reflecting the emerging understanding of natural immunity to HCV, vaccine development has taken two primary and divergent approaches, attempting to either elicit T-cell responses, usually targeting non-structural viral proteins, or induce neutralizing antibodies against viral envelope glycoproteins. Platforms being evaluated in pre-clinical studies include traditional technologies, such as recombinant proteins, synthetic peptides, and virus-like particles often aimed at eliciting antibody responses, and emerging technologies, including genetic vaccines (both DNA and mRNA) and viral vector vaccine approaches designed to elicit T-cell responses [41]. Whole virus vaccines, either inactivated or live-attenuated, have until recently not been pursued due to limitations of HCV cell culture and, in the latter case, safety precautions [42].

Several major bottlenecks have hindered the progression of HCV vaccine candidates from pre-clinical studies to human efficacy trials. First, the only immunocompetent experimental animal model of HCV recapitulating both acute and chronic infection was the chimpanzee, which precluded large challenge studies and is no longer available [43]. This limitation may be partially mitigated by the recent discovery of animal hepaciviruses closely related to HCV. An immunocompetent rat hepacivirus model has been developed, though its ability to predict protection for HCV remains unknown [44]. Second, there exists no established immune correlate of protection against chronic HCV infection to guide the transition from human immunogenicity to efficacy studies [45]. Finally, studies in human populations most at risk for HCV, primarily people who inject drugs (PWID), are logistically difficult and possible only in a small number of specialized cohorts with sophisticated infrastructure for subject retention and care [46,47].

We will herein review the recently published results of the first efficacy trial of an HCV vaccine [48]. This landmark trial tested a promising approach employing viral vectors to encode nonstructural HCV proteins known to generate robust HCV-specific T-cell responses in healthy adults. While this trial, conducted in a cohort of at-risk individuals, confirmed immunogenicity in most vaccine recipients, the immune response elicited was seemingly insufficient to prevent chronic HCV infection. Here, we review the design and results of this study, speculate on potential reasons this vaccine candidate may have failed to protect at-risk individuals, and consider important questions to be addressed by future studies. Given the immense barriers that limit opportunities to transition from pre-clinical to efficacy trials in this field, it will be essential to glean as much direction as possible from this trial to inform future studies.

## 2. The Adenovirus/MVA HCV Vaccine Efficacy Trial

In 2019, the preliminary results from the first human efficacy trial of an HCV vaccine were made publicly available, and full results were published in early 2021 [48]. Conducted from 2012–2018 among at-risk PWID at three sites across the United States (ClinicalTrials.gov NCT01436357), this randomized, double-blinded, placebo-controlled (1:1), phase I/II study assessed the safety and efficacy of an immunization strategy that delivered genes encoding non-structural HCV proteins within viral vector constructs. The primary inoculation used a replication-incompetent recombinant chimpanzee adenovirus 3 (ChAd3) vector encoding NS3-NS5B from a natural genotype 1b HCV strain (ChAd3-NSmut1). The booster dose administered 8 weeks later used a modified vaccinia Ankara (MVA) vector expressing the same NS3-NS5B gene cassette (MVA-NSmut). The trial followed 548 volunteers for 20 months (2 months for vaccination and 18 months for follow-up) with an additional 9 months of observation for volunteers who became infected with HCV to distinguish chronic vs. acute resolving outcomes. The primary outcome measure was development of chronic HCV infection, defined as persistent viremia 6 months after initial detection. Complete follow-up was achieved for 152 of 274 (55.4%) volunteers in the experimental arm and 146 of 274 (53.2%) in the placebo arm. Ultimately, 5.1% of participants in both the vaccinated group (*n* = 14) and the placebo group (*n* = 14) developed chronic HCV infection, indicating that immune responses elicited by the vaccine were unable to prevent the establishment of persistent viremia.

These disappointing results were not readily predicted from earlier studies. The use of replication-incompetent adenoviral vectors carrying genes encoding for HCV antigens was supported by a substantial body of pre-clinical and clinical research showing the capacity to generate CD8^+^ and CD4^+^ T cell responses to HCV thought to be similar to those associated with spontaneous control. The approach was shown to be promising in a chimpanzee challenge model by Capone, Folgori, and colleagues in 2006 [49]. The immunization strategy utilized adenovirus type 6 (Ad6) and adenovirus type 24 (Ad24) vectored constructs expressing NS3-NS5B genes of a natural genotype 1b HCV strain with a mutation designed to eliminate NS5B polymerase activity (NSmut), followed by serial doses of plasmid DNA containing the same NS3-NS5b cassette [50]. In a study of vaccinated chimpanzees, HCV-specific CD4^+^ and CD8^+^ T cells that produced IFN-γ and proliferated after antigen stimulation were detected. Cytotoxic CD8^+^ T cell responses were also detected in four of five animals [49]. Subsequent challenge with a heterologous genotype 1a HCV resulted in significantly reduced viremia and rapid viral clearance in four of five animals when compared to control animals [49]. Further development efforts included screening a large number adenoviral strains to identify candidates that readily infect human cells, are highly immunogenic, and yet rare in humans and thus unlikely to be inhibited by pre-existing immunity [51]. These efforts identified chimpanzee adenovirus 3 (ChAd3) as a promising vector candidate. Human studies in healthy volunteers primed with the ChAd3 vector determined that boosting with modified vaccinia Ankara (MVA) vectored construct resulted in superior CD8^+^ and CD4^+^ HCV-specific T cell responses than did boosting with a second adenoviral construct (Ad6) [52,53]. In phase I trials, all recipients of the ChAd3-NSmut1/MVA-NSmut regimen developed T cell responses against all nonstructural HCV proteins from genotype 1b viruses. CD4^+^ and CD8^+^ T cells produced IFN-γ, TNFα, or IL-2, with many being multi-functional. Using an MHC-class I pentamer, the majority of HCV-specific CD8^+^ T cells were found to have a CD45RA^-^CCR7^-^ effector memory phenotype at the end of the study [53]. A degree of T-cell cross-reactivity was demonstrated to non-genotype 1b peptides, though the response frequency was lower for subtype 1a peptides than 1b, and markedly reduced for distinct genotypes 3a and 4a. Nonetheless, the data were encouraging enough to move the vaccine to phase II efficacy trials.

The lack of efficacy against chronic HCV infection in the phase II trial, despite the promising data from the earlier phase I trial, is perplexing. In the phase II trial, immunogenicity was evaluated within 14 days after the last vaccination by ELISpot, when possible. Of the experimental group participants tested, 77.6% developed measurable IFN-γ producing T cells targeting at least one of six HCV genotype 1b peptide pools [48]. Detailed analyses of the strength, breadth, polyfunctionality, phenotype, and durability of these responses in this larger cohort have not yet been published. Likewise, no information is available regarding T cell cross-reactivity against heterologous viral genotypes. More in-depth analysis of samples from this trial could provide important insight into why this vaccine strategy was ineffective and how future approaches may be adjusted to optimize opportunities for success.

Until additional detailed studies can be conducted, there are still potential insights to be gained from the data available. In the following sections, we consider factors that may, at least in part, help to explain the failure of this vaccine to protect against chronic HCV despite promising immunogenicity, and explore three questions that should be addressed in future vaccine research efforts.

1. **Must an HCV vaccine better address HCV genetic diversity?** The vast genetic diversity of HCV has long been recognized as a challenge for vaccine development. While it was hoped that a vaccine based on a single viral genotype could generate protective cross-reactive responses against a wide variety of genotypes, successful vaccines may require more directed efforts to represent viral antigenic diversity.
2. **Does an HCV vaccine need to elicit neutralizing antibodies?** While it is established that a robust cell-mediated immune response targeting primarily non-structural HCV proteins is strongly linked with spontaneous control, mounting evidence also supports an important role of neutralizing antibodies targeting HCV envelope proteins. Strategies to induce broadly neutralizing antibodies against diverse viral populations continue to advance. A successful vaccine may require the incorporation of both structural and non-structural protein targets to elicit robust neutralizing antibodies and T cell responses that cooperatively avert chronic HCV infection.
3. **Do factors unique to populations at risk for HCV need to be considered in early immunogenicity trials?** Injection drug use is the primary risk factor for HCV acquisition in high-income nations, and PWID are prime candidates for any successful prophylactic HCV vaccine, if approved. Accordingly, active injection drug use was among the inclusion criteria in the ChAd3/MVA-NSmut vaccine efficacy trial. Several factors exist by which immunity and response to vaccination may be altered in PWID, including opiate use and repeated sub-infectious HCV exposure. These factors need to be considered in future HCV vaccine development and trial design.

Finally, though DAA therapy has revolutionized the management of chronic HCV, treated patients do not develop protective immunity after cure and remain vulnerable to reinfection if re-exposed. These individuals will also be important candidates for HCV vaccination. A better understanding of the residual immune defects following cure of chronic HCV infection and how or if new vaccine platforms can overcome this damage to establish protective immunity will be of critical importance for the ultimate elimination of HCV.

## 3. Addressing Genotypic Cross-Reactivity of T Cells

The genetic diversity of HCV exceeds even that of HIV [54,55]. HCV isolates are classified into seven major genotypes and over 90 subtypes. Variability at the nucleic acid level reaches up to 30% between genotypes [56]. While the resolution of infection with one genotype can protect against other genotypes [57], this protection is not absolute [58,59,60]. Indeed, T cells arising during infection target viral epitopes that tend to be genotype- and even subtype-specific. Within a cohort of individuals infected with genotype 3 HCV, very few genotype 1 cross-reactive epitopes were identified [61].

Whether a lack of T cell cross-reactivity contributed to the failure of the genotype 1b ChAd3/MVA-NSmut vaccine to prevent chronic HCV is at this time unknown. In secondary analysis, the vaccine lacked signal of protective efficacy for both genotype 1 and non-genotype 1 infections, precluding any meaningful comparison of cross-genotype protection [48]. IFN-γ ELISpot responses to genotype 1b peptide pools were detected in approximately three-quarters of vaccinated individuals, but responses to other viral genotypes have not been assessed [48]. Likewise, viral sequence data have not yet been assessed to determine the relatedness of infecting and vaccine sequences at relevant CD8^+^ and CD4^+^ T cell epitopes.

Assessments of cross-reactivity of T cell responses elicited by the genotype 1b ChAd3/MVA-NSmut vaccine are available from phase I immunogenicity trials in healthy control subjects. Indeed, cross-reactive IFN-γ ELISpot responses were detected against peptide pools from heterologous genotypes, but with reduced frequency. Compared to the robust responses to genotype 1b peptides, T cell reactivity was reduced by approximately 40% against closely related subtype 1a peptides, and by 70% against more divergent genotypes 3a and 4a [53]. Whether these responses would be sufficient to protect against persistent infection upon heterologous virus challenge is unknown [53]. Despite remaining uncertainties as to whether deficient cross-reactivity contributed to the failure of the ChAd3/MVA-NSmut vaccine phase II trial, it is reasonable to expect that future vaccine iterations should incorporate antigen designs that generate more broadly reactive responses across genotypes.

Numerous vaccine approaches to the challenge of viral diversity are being evaluated for HCV and other viruses including HIV. One such approach aims to specifically target regions of the virus that are conserved across multiple viral clades or genotypes by stitching together conserved regions and excluding highly diverse regions of the viral proteome. This has been pioneered in the development of new HIV vaccine candidates. In the case of the HIV_CONSV_ vaccine, a chimeric protein was developed using conserved regions from HIV-1 clades A, B, C, and D with the goal of developing T cell responses to epitopes that are more likely to be shared among a multitude of strains [62]. The HIV_CONSV_ regions appear to be widely targeted in individuals with natural HIV infection, though, interestingly, the responses to the conserved epitopes commonly represent subdominant T cell responses [62]. This construct has been shown to be immunogenic in uninfected mice [62], macaques [63], and humans [64,65]. While using this approach has shown only partial protection in macaques to date [66], the data have been promising and efforts to apply a similar approach in novel HCV vaccines are underway [67,68]. Rather than use conserved genetic regions from various HCV genotypes, von Delft and colleagues used circulating HCV isolates that had the highest homology to the consensus sequences of conserved genetic regions in each group of viral isolates of interest (genotypes 1a/b, genotypes 1/3, and genotypes 1–6) as a starting point. The group then stitched together conserved regions and removed the variable regions. This yielded three new vaccine vectors for targeting different sets of HCV genotypes, including one vector meant to target all but genotype 7 viruses, dubbed GT1-6L [67]. Initial tests of GT1-6L, encoded in a chimpanzee adenovirus ChAdOx1, demonstrated immunogenicity in mice. ELISpot assays also showed that GT1-6L elicited T cell response to peptides from genotype 1a, 1b, and 3a viruses at similar levels [67]. Further study demonstrated that immunization of mice with ChAdOx1-GT1-6L induced both a higher magnitude and wider breadth of T cell responses against genotype 1b peptide pools when compared to ChAdOx1-GT1b-NS, a control vaccine based on the previous first-generation vaccine [68]. GT1-6L also elicited better T cell responses in terms of both magnitude, when splenocytes were stimulated with genotype 3a peptides, and breadth, when stimulated with genotype 1a peptides [68]. While safety and immunogenicity results in non-human primates and humans are not yet available, the current data suggest that this approach to generating pan-genotypic T cell responses is promising.

A separate approach to minimize sequence dissimilarity between a candidate HCV vaccine antigen and contemporary circulating HCV strains is the rational design of Mosaic or Epigraph antigens. Mosaic and epigraph antigens are designed computationally by recombination of viral genomic sequences retrieved from databases, with the requirement that all recombination breakpoints exist in natural HCV sequences, precluding the creation of artificial junctional epitopes [69,70,71]. Viral diversity is overcome in two ways. First, less common amino acids are disfavored at each position of the antigen, reducing the probability of vaccine- or strain-specific T cell responses, while common amino acids are favored to maximize the most common potential T cell epitopes [69,70,71]. Second, these vaccines are typically polyvalent to further expand the breadth of epitope and maximize sequence diversity. Importantly, mosaics and epigraphs are indistinguishable from natural viral proteins in sequence and processing for class I and class II presentation. Mosaic design is the most advanced for HIV vaccines [72]. In macaques, HIV-1 mosaics induced significantly higher numbers of cross-reactive CD4^+^ and CD8^+^ T cells than natural protein vaccines [73,74]. Vaccinated animals were also more difficult to infect and did not succumb to infection when compared to unvaccinated macaques [75]. Mosaic HIV vaccines have demonstrated immunogenicity in humans [76,77], suggesting potential for translation to an HCV vaccine. The feasibility of mosaic design of a pan-genotypic HCV vaccine has been demonstrated by in silico analysis, with multivalent mosaic antigens providing the best coverage against diverse HCV isolate sequences [54]. Immunization experiments further demonstrated the immunogenicity of mosaic HCV vaccines in mice and found that they induced more robust responses than immunization with antigens from natural viral strains [78]. Further iterations on the HCV mosaic/epigraph vaccine platform involving the incorporation of different delivery platforms, new adjuvants, and boosting strategies, and further murine studies will be required before transitioning to non-human primates for continued safety and immunogenicity studies.

While both the conserved-region vaccine and mosaic/epigraph vaccine approaches offer their own advantages and have promise individually, using a combination of methods may yield the most effective result. Indeed, in the field of HIV, a new generation of HIV_CONSV_ vaccines, HIV_CONSV_X, has been introduced. These second-generation vaccines incorporate both the use of conserved regions and computationally developed Mosaic approaches in a multivalent vaccine, and they have been shown to be highly immunogenic [79], with some components being currently evaluated in phase I clinical trials (NCT03844386). Because circulating HCV may demonstrate wider variability than HIV, similarly multi-pronged approaches to developing a T cell-focused HCV vaccine may also prove necessary.

## 4. Harnessing Neutralizing Antibody Responses in New Vaccines

While multiple lines of evidence support a central role of CD8^+^ and CD4^+^ T cells in the control of HCV infection, the role of antibodies in resolving infection and, by extension, their potential protective value in vaccine-elicited responses, has been less clear. Documented cases of HCV clearance in seronegative PWID and in patients with agammaglobulinemia [29,30,31] indicated that antibodies may be dispensable. To address whether humoral responses may yet contribute to viral clearance in some patients, multiple studies have attempted to identify correlations between infection outcome and the development of antibodies, particularly neutralizing antibodies that target the HCV envelope glycoprotein heterodimer responsible for viral attachment and entry, E1/E2. Early studies that measured neutralization of lentiviral pseudoparticles expressing HCV E1E2 glycoproteins (HCVpp) from prototypical HCV strains such as H77 found high titer serum neutralizing antibodies during the chronic phase of infection and absent or rare antibodies in early infection that did not associate with viral clearance [32,33]. In contrast, several studies that used HCVpp bearing autologous E1E2 sequences found viral clearance to be associated with rapid induction of neutralizing antibodies against the transmitted or early circulating viral strain [39,80,81,82]. A study using a library of HCVpp with representative E1E2 sequences linked spontaneous viral clearance with the early development of broadly neutralizing antibodies [40]. Moreover, E2-specific memory B cells have been found to expand and peak more rapidly in resolvers than those who progress to chronic infection. This rapid expansion is associated with more robust early activity of circulating IL-21 producing T follicular helper (Tfh) cell populations [83]. Thus, an increasing preponderance of evidence suggests that the rapid development of specific and broadly neutralizing antibodies in acute infection may contribute to viral clearance.

Although this understanding of the role of antibodies in HCV infection outcome has taken some time to come into view, neutralizing antibodies have been a focus of HCV vaccine development efforts from the outset given their importance in vaccination strategies against other viral infections [38]. An early construct consisting of purified recombinant HCV genotype 1a (strain HCV-1) E1E2 protein administered to chimpanzees with an oil/water emulsion adjuvant elicited E1E2 antibodies that in some cases appeared to confer sterilizing immunity upon challenge with homologous virus HCV-1 virus [37] and protected against chronic infection upon challenge with a heterologous genotype 1a H77 strain [38]. The overall rate of chronic infection in 31 E1E2 vaccinated chimpanzees challenged with genotype 1a viruses was 16%, compared to 63% in 24 unimmunized controls [38,84]. Serum from E1E2-vaccinated chimpanzees exhibited cross-neutralization of HCVpp or cell-cultured HCV (HCVcc) expressing envelope proteins of other genotype 1a viruses as well as genotypes 4, 5, and 6, but had little activity against genotypes 2 or 3 [85]. Given the promising pre-clinical data, the recombinant HCV-1 E1E2 construct adjuvanted with MF59 proceeded to phase I trials in healthy volunteers [86]. The vaccine was well-tolerated and reliably elicited high titers of E1E2-binding antibodies and robust proliferative CD4^+^ T helper responses [86]. Serum from most vaccinees could interfere with E2 binding to the CD81 receptor and neutralize HCVpp or HCVcc expressing autologous HCV-1 E1E2 [86,87] but rarely had broad cross-genotype neutralization activity [87,88].

Follow-up studies found the most dominant responses elicited by E1E2 vaccination targeted the N-terminus of E1 and the hypervariable region 1 (HVR1) at the N-terminus of E2 and were mostly non-neutralizing [87]. Neutralizing antibodies were raised against HVR1 as well as conserved sites associated with the potential for broad cross-reactivity such as the antigenic region 3 (AR3) located on the “neutralization face” of E2 that overlaps the CD81 binding site [87]. AR3-specific broadly neutralizing antibodies have been associated with HCV clearance and commonly utilize a particular immunoglobulin heavy-chain-variable gene *V_H_1-69* [89,90,91]. These AR3-specific *V_H_1-69-*encoded broadly neutralizing antibodies often have quite limited somatic hypermutation, suggesting that a germ-line targeting vaccine approach to elicit this desirable response may be feasible [92]. E1E2 immunized rhesus macaques also generated AR3-neutralizing antibodies that had limited somatic hypermutation. These utilized a heavy chain variable gene *V_H_1-36* that is homologous to human *V_H_1-69*, potentially providing a pre-clinical model for germline-targeting vaccine strategies [87,92].

Together, studies of the recombinant HCV-1 E1E2 vaccines provide a solid basis for the pursuit of an antibody vaccine, while highlighting the need for optimization to better elicit robust neutralizing responses against conserved regions of the envelope glycoprotein. As such, significant effort has been made to engineer a rational antibody vaccine for HCV, as recently reviewed in detail by several groups [93,94,95]. These efforts follow insights gained from crystal structures of antibody-bound constructs of truncated E2, improved modeling of the E1E2 heterodimer, and expanded mapping of neutralizing and non-neutralizing antibody binding sites on E1E2. One proposed approach has been to delete highly variable regions such as HVR1, which are purported to act as immunodominant decoy antigens, readily evading strain-specific antibodies by mutational escape while simultaneously interfering with responses to more conserved regions [96,97,98,99,100]. Deletion of HVR1 from adjuvanted E1E2 vaccine reduced the development of homologous strain-specific neutralizing antibodies while also failing to enhance heterologous responses in mice [101]. However, deletion of HVR1 along with other variable regions including HVR2 and VR3 from core E2 structures led to a stabilized E2 structure with preserved “neutralizing face” [102,103] that, when delivered in oligomeric form, induced robust pan-genotypic neutralizing response in guinea pigs and fewer non-neutralizing antibodies [104]. Analogous E2 constructs lacking HVR1 and VR3 with a modified VR2 and incorporated into nanoparticles also elicited robust pan-genotypic responses in rodents [105]. Beyond shielding by the immunodominant HVR1 region, conserved epitopes have been shown to be shielded from immune recognition by glycosylation across E1 and E2 [106,107,108]. Removal of select glycosylation sites from E1E2 immunogens has been shown to improve the production of neutralizing antibodies and to enhance cell-mediated responses [109,110]. Manipulation of E2 glycosylation by expression in non-mammalian cells may also accentuate immunity to conserved neutralizing epitopes [111]. Finally, beyond uncovering conserved sites, mosaic E1E2 immunogens may also present an approach to the diversity of HCV envelope antigens. While current efforts in the development and testing of mosaic HIV vaccines focus on cell-mediated immunity, they also elicit neutralizing antibodies that target the variable envelope protein of HIV [69,73,77]. Immunization with a mosaic HCV E2 immunogen has shown enhanced immunogenicity in mice, though neutralizing antibodies were not specifically studied [78].

### New Vaccine Delivery Platforms and a Unified Approach to Target T Cell and B Cell Responses

A vaccine regimen that elicits both broadly neutralizing antibodies and potent broad CD8^+^ and CD4^+^ T cell responses may have the best odds of preventing chronic HCV infection. While this seems an obvious vaccine objective, design of such a vaccine requires consideration of both antigen selection and delivery platform. E1E2 or E2 alone is the required immunogen for generation of neutralizing B cell responses, but non-structural proteins may be better immunogens for T cell responses, as they are more commonly targeted by T cells in natural infection and more conserved (e.g., NS3 and NS5B) than envelope glycoproteins [112]. In terms of vaccine platforms, recombinant protein antigens are well-suited for induction of B cell responses with follicular CD4 T cell help, while intracellular expression and processing of vaccine proteins such as with viral-vectored or DNA vaccine platforms is optimal for MHC I loading to induce CD8^+^ T cell responses. Thus, one approach would be a regimen combining two different vaccines, one with an envelope protein immunogen in a platform for B cells, and a second with a non-structural protein immunogen delivered in a platform for optimal CD8^+^ T cell responses. Alternatively, advances in vaccine adjuvant, immunopotentiator, and vaccine particle development offer the potential to trigger multiple immune pathways with a single vaccine platform, such as recombinant protein antigens that with certain adjuvants not only trigger robust B cell and CD4^+^ T cell responses but also induce cross-presentation to CD8^+^ T cells. Numerous such platforms are being actively evaluated in pre-clinical and limited clinical studies of new HCV vaccines, and recent reviews have examined this in more comprehensive detail [113,114].

The recent success of mRNA vaccines encapsulated in lipid nanoparticles (LNPs) for SARS-CoV-2 [115,116] raises another intriguing option for an HCV vaccine that might induce protective antibody and T cell responses. The BNT162b2 and mRNA-1273 SARS-CoV-2 vaccines both encode a full-length, pre-fusion stabilized SARS-CoV-2 spike protein and are aimed at eliciting neutralizing antibodies that prevent interactions between the viral spike protein and the cellular receptor ACE2 [117,118]. Both vaccines elicit high neutralizing anti-spike antibody titers as well as polyfunctional antigen-specific CD4^+^ T cells with a significant skew towards Th1 responses and away from Th2 responses in humans and non-human primates [117,119,120,121]. In non-human primates, the vaccines have also been shown to elicit populations of antigen-specific Tfh populations that may provide essential help to B cells in the germinal centers of lymph nodes [117,120]. Despite the many similarities, only BNT162b2 has been observed to elicit robust antigen-specific CD8^+^ cytotoxic T cell populations, whereas similar populations are limited to undetectable after mRNA-1273 administration [117,119,120,121]. The potential for these mRNA-based vaccine platforms to elicit both robust antibody and T cell responses may be incredibly promising for a novel HCV vaccine candidate, but while the selection of the best antigens will be crucial as previously discussed, the lack of CD8^+^ T cell responses to mRNA-1273 highlights that other components, including the composition of the LNP, may also be essential for eliciting the desired immune responses with such an approach.

## 5. Understanding Vaccine Immunogenicity in Populations Most at Risk for HCV

The ChAd3/MVA-NSmut HCV vaccine elicited robust polyfunctional HCV-specific T cell responses in early phase I immunogenicity trials, making its lack of protection from chronic infection in the phase I/II efficacy trial puzzling. As discussed in the preceding sections, a successful HCV vaccine will likely need to better address viral diversity and potentially incorporate humoral responses to envelope glycoproteins. Another consideration is that the quality of T cell responses in phase II vaccinees may not have matched the robust responses found in earlier immunogenicity trials. Indeed, the cursory data on immune responses provided with the phase II study suggests that this is the case. Whereas 100% of phase I participants had IFN-γ ELISpot responses to ≥4 of 6 HCV peptide pools spanning HCV proteins NS3-NS5 [53], only 78% of phase II participants had a measurable response to any peptide pool [48]. Similarly, the combined magnitude of peak HCV-specific IFN-γ responses after the 2nd dose of vaccine was substantially lower in the phase II trial (median 428 spot-forming cells per million PBMC in phase II trial versus 2355 in the phase I study).

Reasons for the discrepant immunogenicity results are not yet known, but study population differences may have contributed, particularly with regard to injection drug use [48]. The phase II clinical trial specifically enrolled participants with a known recent history of injection drug use, while earlier phase I immunogenicity studies had excluded individuals with suspected or known injection drug use [53]. The phase II trial established the feasibility of working with PWID in a vaccine clinical trial, an important accomplishment for future HCV vaccine efficacy studies. However, it is possible that injection drug use may have adversely affected immune responses to the HCV vaccine, through mechanisms such as immunosuppressive effects of opioids or damping effects of repeated subinfectious exposure to HCV prior to vaccination, as discussed below. Fully appreciating and accounting for the immune effects of injection drug use will be important for future vaccine trials, particularly those in which PWID are a key high-risk group who could significantly benefit from prophylactic vaccines.

### 5.1. Opioid Usage and Effects on Immunity and Vaccines

An ongoing opioid epidemic has fueled the surge in acute HCV cases in the United States over the last two decades [122]. While the strong association between opioid use and HCV infection is mediated principally by viral transmission via shared injection paraphernalia, there has been some concern that opioids may also adversely affect immunity and thus increase susceptibility to HCV and potentially impair HCV vaccine responses. Opioid use has been linked to heightened risk of other infectious diseases. including HIV, TB, and pneumonia, and to a higher risk of mortality in sepsis patients [123,124]. Suboptimal seroconversion rates to HBV vaccine have been noted among individuals injecting heroin compared to healthy adult populations [125]. Supporting these clinical observations, numerous rodent and non-human primate animal model studies, as well as ex vivo human studies, have documented acute and chronic opioid exposure as being associated with impaired bulk T and B cell mitogen responses, depressed bulk CD4/CD8 ratios, and altered T helper differentiation away from Th1 responses in bulk populations [126,127,128,129,130,131,132,133]. Innate immune effects include impaired NK cell toxicity and depressed phagocytic activity of monocytes and macrophages [134]. The effects of opioids on immunity may be mediated in part via neuroendocrine or neuroimmune axes, but direct effects on immune cells are also suggested given that defects can be recapitulated with opioid treatment of isolated cells in vitro. This is further supported by the detection of opioid receptor expression by numerous immune cell subsets, particularly following cell activation [135].

A recent study examined the effects of opioid exposure on antiviral gene expression in human PBMCs by single-cell RNA-seq. Following LPS stimulation, PBMCs from individuals with chronic opioid use, and PMBCs from healthy individuals treated with morphine in vitro demonstrated reduced interferon-stimulated gene and antiviral gene expression compared to untreated healthy control PBMCs. This effect was observed in all identified cell subsets including monocytes, CD4^+^ T cells, CD8^+^ T cells, B cells, and NK cells [136]. Interestingly, morphine treatment of hepatocytes has also been associated with reduced IFN-α production and enhanced HCV replication in vitro [137]. This inhibition of innate defenses may indicate that, regardless of additional inhibition of any adaptive immune functions, opioid users could be more susceptible to HCV infection and a vaccine response may need to be particularly robust to effectively prevent chronic infection.

Despite the long history of experimental and epidemiologic evidence supporting an immunosuppressive effect of opioids, results have been inconsistent. Some studies have failed to identify in vitro or in vivo effects of opioids on immune cell function. While discrepancies may be in part related to the dosing regimen or type of opioid studied, a more complex relationship between opioids and immunity is likely at play, with recent studies noting an immune-activating effect of chronic opioid exposure [138,139]. Additionally, numerous vaccine immunogenicity studies failed to identify an effect of opioid use on seroconversion [140,141,142]. Further study of the ChAd3/MVA-NSmut vaccine cohort presents a unique opportunity to explore the effects of injection drug use on a vaccine targeting a T cell response, if samples obtained during the phase I/II clinical trial can be compared via detailed functional and phenotypic studies to samples obtained during early immunogenicity trials in healthy controls [53].

### 5.2. Effects of Sub-Infectious Exposure to HCV on Adaptive Immunity

While PWID are at a high risk of acquiring HCV due to exposure to infectious doses of HCV via needle sharing, evidence suggests that this group also frequently encounters minute viral doses that do not lead to sustained viremia and seroconversion. Indeed, a high percentage of HCV RNA-negative seronegative PWID with detectable populations of HCV-specific T cell populations targeting non-structural proteins have been observed in multiple studies [31,143,144,145]. Sub-infectious HCV exposure associated with HCV-specific T cell responses and a lack of antibody response has also been documented in other groups, including healthcare workers [146,147,148,149], family members of chronically infected patients [150,151,152], and sexual partners of patients with acute infection [153]. An important caveat is that many of these studies cannot definitively rule out the possibility of previous resolution of acute infection leading to HCV-specific T cell responses with a loss of detectable antibodies over time [154]. However, prospective studies that followed cohorts of healthcare workers after HCV exposure through accidental needlesticks demonstrated that subsequent development of HCV-specific T cell response without detectable antibodies does occur [147,148].

Low-dose sub-infectious exposures and the development of T cell responses have been hypothesized to offer some level of immune protection against future infectious exposures to the virus. While this has been difficult to definitively prove with cross-sectional and prospective studies in humans, a study using the chimpanzee model of HCV infection directly assessed this hypothesis [155]. In this study, chimpanzees were given multiple infusions of plasma and an infusion of PBMCs from patients who had trace amounts of HCV that were below the limit of detection of clinical assays. Two animals that did not develop detectable viremia developed HCV-specific T cells responses and, when subsequently challenged with infectious doses of HCV, developed significantly higher and sustained viremia with weaker HCV-specific CD4^+^ and CD8^+^ T cell responses when compared to a chimpanzee that was infected after previously resolving acute HCV infection [155]. Control chimpanzees that received a mock treatment prior to challenge showed a more robust T cell response after a viral challenge compared to those that were pre-exposed to low viral doses. The authors examined bulk CD4^+^ Treg cells in the blood and found that the frequency was significantly higher in chimpanzees that were pre-exposed to HCV, and bulk Treg populations expanded more in this group after viral challenge in comparison to control animals. Depletion of these Tregs in vitro resulted in a more significant increase in HCV-specific T cells responses in IFN-γ ELISpot assays compared with the depletion of Tregs from control animals, suggesting that the expansion of bulk Treg population contributed to a hampered immune response to HCV challenge after low-dose pre-exposure [155].

The phase I trial of the ChAd3/MVA-NSmut vaccine listed recent injection drug use as an exclusion criterion. HCV-specific T cell responses were not detected in any participants at the onset of the study, suggesting that participants had not been exposed to even low doses of HCV [53]. Vaccinated participants were shown to have bulk Treg frequencies similar to unvaccinated healthy volunteers [53]. In contrast, the phase I/II trial required the involvement of participants at high risk of HCV exposure, namely PWID [48]. Although HCV-specific T cell responses were assessed prior to enrollment and only 3.3% of evaluated participants in the placebo group were indicated to have HCV-specific T cell responses after receiving the placebo treatment, HCV-specific T cell responses in seronegative PWID have often been observed to be somewhat weak, and responses may be under the limit of detection with the methods utilized [31,144,145,148]. Detailed analysis of samples taken before and after vaccination, particularly as it pertains to the frequency of bulk or antigen-specific Treg cells, will be important to determine if potential prior sub-infectious exposure could have had any effect on vaccine immunogenicity. Additionally, the potential influence of sub-infectious exposure further suggests that, with a vaccine that will be chiefly important in populations of PWID, involving this population early in phase I safety and immunogenicity trials will be needed to ensure any new vaccine candidate is in fact immunogenic in the target population prior to larger and more complex trials. If immunogenicity in this population is found to be muted, whether due to opioid use or previous sub-infectious exposures, the addition and use of novel adjuvants or vaccine platforms to enhance immunogenicity in PWID may be required. For example, the inclusion of MHC class II-associated invariant chain in a viral vectored HCV vaccine broadly enhanced CD4^+^ and CD8^+^ T cell responses over the original vaccine in recently conducted human clinical trials [156].

### 5.3. Impact of Chronic Infection and DAA Therapy on Potential Vaccine Success

Unlike spontaneous resolution of acute HCV infection, cure of chronic HCV infection with DAA therapy does not appear to lead to protective immunity. Reinfection has been described in numerous human cohort studies following successful treatment with DAA or interferon-α/ribavirin therapies [12,157,158], and among individuals with opioid addiction, the risk of reinfection is tied to continued use of injection drugs. Consequently, opioid-agonist therapy before or after DAA treatment significantly reduces the risk of reinfection [12,159]. Unfortunately, those who are cured with DAA therapy and subsequently reinitiate injection drug use, if reinfected, can reestablish themselves as reservoirs of HCV and, with continued high-risk behavior, unintentionally infect new people. Curing PWID of HCV and subsequently mitigating the risk of reinfection, through a combination of reducing high-risk behavior and the development of protective immunity, will be an essential component of eliminating HCV.

The lack of protective immune memory against reinfection following DAA cure suggests that immune defects that occur during chronic infection are not fully reversed after the virus is cleared. Major features of the adaptive immune response during chronic infection include loss of HCV-specific CD4^+^ T cell help [17,18,26,160,161,162] and failure of the HCV-specific CD8^+^ T cell response due to exhaustion [163,164,165] or emergence of viral escape mutations that prevent the recognition of CD8^+^ T cell epitopes [166,167,168,169]. Studies done since the introduction of DAA therapies have begun to shed light on how immunity continues to change after recovery from viral infection and, importantly, how this new immune state is markedly different from that which is observed after spontaneous resolution of infection. It has been observed that HCV-specific CD8^+^ T cell populations expand following DAA-mediated viral clearance [170,171], though in a chimpanzee study, this expansion occurred preferentially in CD8^+^ T cells that were previously dominant and had targeted escaped epitopes [172]. In human studies, expanded HCV-specific CD8^+^ T cell populations observed post-DAA cure have a better capacity to proliferate and produce antiviral cytokines than prior to treatment, though these cells are neither as proliferative nor as functional as those observed in acute resolving infection [171,173]. These cells are a memory-like T cell subset expressing CD127, PD-1, and the transcription factor TCF1 and are distinct from more conventional memory cells that are observed after spontaneous viral clearance. If re-exposed to antigen, the “exhausted memory-like” subset may differentiate into a terminally exhausted subset that are CD127^-^PD-1^high^TCF1^-^, a subset of cells that were present during initial chronic infection and lost after DAA-mediated viral clearance [173]. Recent detailed transcriptional profiling of these HCV-specific CD8^+^ T cell subsets before and after DAA-mediated HCV cure has reinforced the distinct nature of these cells when compared to the memory populations that develop after spontaneous resolution of acute infection [174]. The reduced functionality of HCV-specific CD8^+^ T cells is possibly linked with dysfunctional metabolism that is observed in chronic infection and maintained after DAA therapy [175,176,177]. Additionally, TOX, a transcription factor linked to transcriptional and epigenetic reprogramming of CD8^+^ T cells and the development of T cell exhaustion has been found to be highly expressed in HCV-specific CD8^+^ T cells during chronic infection and after DAA-mediated viral clearance but not after spontaneous resolution of infection [178]. Comparatively less is known about the HCV-specific CD4^+^ T cells both during and after chronic HCV infection due to the very low frequency of these cells in peripheral blood after the initial establishment of chronicity. Despite this challenge, several recent studies have demonstrated that the HCV-specific CD4^+^ T cell population increases in frequency at least transiently in the peripheral blood during and after DAA treatment [179,180]. However, in a study comparing samples from treated patients and vaccinated subjects from human trials, this increase in frequency was not observed [181]. These conflicting results may be explained by differing sampling timelines. Regardless, the frequency of HCV-specific CD4^+^ T cells after DAA treatment is not likely to be comparable to frequencies observed in spontaneous clearance [181]. During and after DAA treatment, HCV-specific CD4^+^ T cells exhibit a decrease in expression of some, but not all, inhibitory receptors, including PD-1, as well as a decrease in the expression of numerous activation markers. Additionally, these cells seem to shift toward a memory phenotype with increased CD127 and TCF1 expression and, strikingly, a shift from a Th1 phenotype to a predominantly follicular helper T cell (Tfh) phenotype [179,180]. The ramifications of these phenotypic shifts are not yet fully understood. Collectively, it is clear that chronic HCV infection leaves a lasting imprint on the HCV-specific adaptive immune response even after viral eradication.

In addition to HCV-specific immune exhaustion that occurs as acute infection proceeds to chronicity, some individuals cured of longstanding infection have residual advanced liver fibrosis or cirrhosis that may itself impair innate and adaptive immunity [182,183] and potentially hinder HCV vaccine responses. For instance, relative to HCV-infected individuals with minimal liver disease (F0-1), those with cirrhosis (F4) have a significant shift of generalized CD8^+^ T cell phenotype and function towards a hyperfunctional cytotoxic state, and this state persists after DAA cure [184]. Persistent high levels of serum proinflammatory cytokines were also noted after DAA cure in individuals with advanced liver disease [184]. Of concern for secondary HCV prevention through vaccination, cirrhosis has also been associated with impaired IFN-γ ELISpot T cell response to influenza vaccination [185].

While HCV-naïve individuals are the focus population for current HCV vaccine development efforts, evaluating candidate vaccines and novel adjuvant formulations in individuals cured of HCV by DAA therapy with or without advanced cirrhosis will be an important topic of research. If an HCV vaccine is found to be less immunogenic in this group, coupling the vaccine with new therapies to “reverse” the exhausted phenotype of adaptive immunity may also be a consideration. As an example, recent in vitro data suggest that histone methyltransferase inhibitors or p53 inhibitors may have the ability to restore CD8^+^ T cell functionality and proliferative capacity [176]. With a number of histone methyltransferase inhibitors already in clinical trials for cancer therapy [186], the possibility of repurposing one or more of them for treatment coupled to DAA therapy and/or vaccination to potentially increase the functional capacity of previously exhausted CD8^+^ T cells is intriguing.

## 6. Conclusions

Despite the development of potent DAA therapies, a prophylactic vaccine is urgently needed to eliminate the public health burden of HCV. The recent failure of the ChAd3/MVA-NSmut regimen in the first large-scale clinical trial of an HCV vaccine was unquestionably a considerable disappointment. Nevertheless, data from this trial, along with recent advances in our understanding of HCV immunity and continued advancement in vaccine immunogen design and delivery systems, offer direction for developing and testing the next generation of HCV vaccines.

## Abbreviations

HCV	Hepatitis C Virus
DAA	Direct-acting antiviral
PWID	People who inject drugs
LNP	Lipid nanoparticles
